# Fungal Aflatoxins Reduce Respiratory Mucosal Ciliary Function

**DOI:** 10.1038/srep33221

**Published:** 2016-09-14

**Authors:** Robert J. Lee, Alan D. Workman, Ryan M. Carey, Bei Chen, Phillip L. Rosen, Laurel Doghramji, Nithin D. Adappa, James N. Palmer, David W. Kennedy, Noam A. Cohen

**Affiliations:** 1 Department of Otorhinolaryngology – Head and Neck Surgery, University of Pennsylvania Perelman School of Medicine, Philadelphia, Pennsylvania, USA; 2 Department of Physiology, University of Pennsylvania Perelman School of Medicine, Philadelphia, Pennsylvania, USA; 3 Philadelphia VA Medical Center Surgical Services, Philadelphia, Pennsylvania, USA; 4 Monell Chemical Senses Center, Philadelphia, Pennsylvania, USA

## Abstract

Aflatoxins are mycotoxins secreted by *Aspergillus flavus*, which can colonize the respiratory tract and cause fungal rhinosinusitis or bronchopulmonary aspergillosis. *A. flavus* is the second leading cause of invasive aspergillosis worldwide. Because many respiratory pathogens secrete toxins to impair mucociliary immunity, we examined the effects of acute exposure to aflatoxins on airway cell physiology. Using air-liquid interface cultures of primary human sinonasal and bronchial cells, we imaged ciliary beat frequency (CBF), intracellular calcium, and nitric oxide (NO). Exposure to aflatoxins (0.1 to 10 μM; 5 to 10 minutes) reduced baseline (~6–12%) and agonist-stimulated CBF. Conditioned media (CM) from *A. fumigatus*, *A. niger*, and *A. flavus* cultures also reduced CBF by ~10% after 60 min exposure, but effects were blocked by an anti-aflatoxin antibody only with *A. flavus* CM. CBF reduction required protein kinase C but was not associated with changes in calcium or NO. However, AFB_2_ reduced NO production by ~50% during stimulation of the ciliary-localized T2R38 receptor. Using a fluorescent reporter construct expressed in A549 cells, we directly observed activation of PKC activity by AFB_2_. Aflatoxins secreted by respiratory *A. flavus* may impair motile and chemosensory functions of airway cilia, contributing to pathogenesis of fungal airway diseases.

Mucociliary clearance (MCC) is the primary physical defense of the respiratory tract against inhaled pathogens^1^. When MCC fails, respiratory infections significantly impair patient quality of life^1^. Upper respiratory infections (URIs) often result in chronic rhinosinusitis (CRS), a complex syndrome involving stasis of sinonasal secretions and inflammation. CRS causes ~8 billion dollars of yearly direct healthcare costs in the US alone^2^ and accounts for ~20% of adult antibiotic prescriptions^3^,^4^,^5^, making CRS a major driver of antibiotic resistance^6^,^7^. URIs also “seed” lower respiratory infections and/or exacerbate lung diseases such as cystic fibrosis or chronic obstructive pulmonary disease^8^. It is important to better understand the complex pathogenesis of respiratory infections to generate novel therapies and improve outcomes for these airway diseases.

Fungi of the *Aspergillus* genus are ubiquitously found in nature^9^,^10^. While ~90% of *Aspergillus* are non-pathogenic, some are opportunistic pathogens that colonize the human respiratory tract^9^,^10^, including *A. fumigatus*, *A. niger*, and *A. flavus*. Sensitization to colonizing *Aspergillus* can cause allergic bronchopulmonary aspergillosis^11^ or allergic fungal rhinosinusitis^12^. Moreover, in immunocompromised individuals (e.g., chemotherapy patients) or those with damaged airways (e.g., CF or CRS patients) *Aspergillus* infections can invade the mucosa^9^. Acute or chronic invasive fungal rhinosinusitis or pulmonary aspergillosis requires aggressive antifungal and/or surgical intervention to prevent mortality. It is particularly critical to understand the interactions of *Aspergillus* with the airway epithelium to understand the pathogenesis of fungal respiratory infections.

Many respiratory pathogens secrete factors that impair MCC, including *Pseudomonas aeruginosa*^13^,^14^,^15^ and *Streptococcus pneumoniae*^16^. The fungus *A. fumigatus* also secretes gliotoxin, fumagillin, and helvoilic acid^10^,^17^,^18^,^19^, which can slow ciliary beat frequency (CBF)^18^. The mechanism(s) of their ciliotoxicities are not yet well defined, but conditioned media (CM) from clinical isolates of *A. fumigatus* as well as sputum from patients with pulmonary aspergillosis impair cilia function and damage epithelial tissue^17^,^18^. While *A. fumigatus* is the most common pathogenic *Aspergillus*, *A. flavus* is the second-leading cause of invasive aspergillosis^20^,^21^. *A. flavus* infection is rare in the US and Europe. However, bronchiopulmonary and sinonasal apergillosis from *A. flavus* is common in India, Africa, South East Asia, and the Middle East, possibly due to an increased ability of *A. flavus* to thrive in arid conditions^20^. *A. flavus* in the upper respiratory tract is often associated with chronic granulomatous sinusitis. *A. flavus* is of importance because it produces aflatoxins, which are among the most potent naturally-occuring hepatic carcinogens known^19^. Ingestion of contaminated foods results in metabolism (“activation”) of aflatoxins in the liver into reactive DNA-damaging epoxides that cause hepatic necrosis, cirrhosis, and/or carcinoma^22^,^23^.

Inhalation of aflatoxins has been associated with occupations involving exposure to environmental molds^24^, such as grain processing. However, the effects of inhaled aflatoxins or aflatoxin-producing fungi on the airway epithelium are not well characterized. There is some evidence that airway cells can activate aflatoxins *in vitro*^4^,^25^ and *in vivo*^26^,^27^,^28^, though the link between aflatoxin exposure and human lung cancer is unclear. However, aflatoxins can increase protein kinase C (PKC) activity in some cell lines *in vitro*^29^,^30^,^31^. Because PKC can decrease CBF^32^,^33^ through phosphorylation of ciliary proteins^32^,^33^, we hypothesized that aflatoxins may have acute effects on MCC that contribute to *A. flavus* pathogenesis.

## Results

### Aflatoxin B_2_ Decreases CBF in a PKC-Dependent Manner

**Figure 1:**
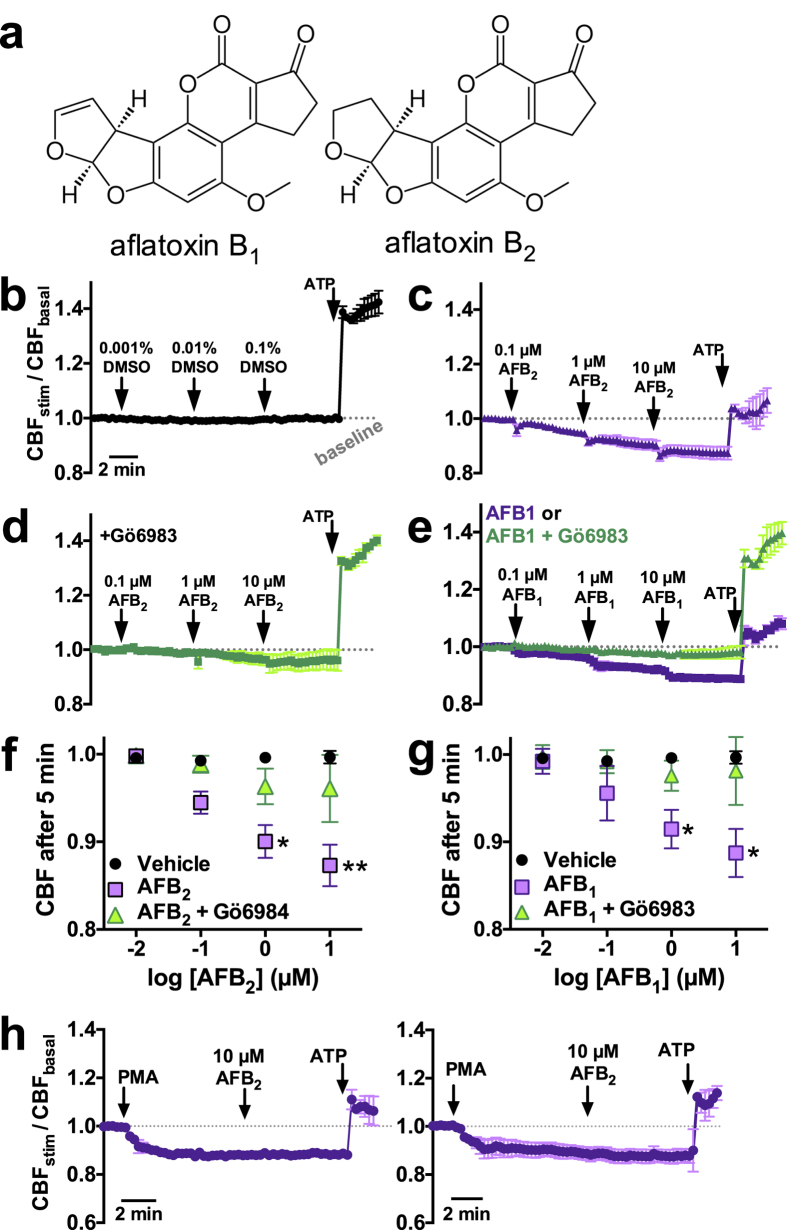
Acute exposure to AFB_2_ and AFB_1_ decreased basal sinonasal CBF in a PKC-dependent manner. (**a**) Structures of aflatoxins B_1_ and B_2_ (AFB_1_ and AFB_2_). (**b–e**) Mean traces of CBF normalized to baseline (n = 3–6 cultures from separate patients each) during stimulation with vehicle (DMSO) alone (*b*), AFB_2_ (5 min exposure for each concentration; *c*), AFB_2_ + Gö6983 (*d*), AFB_1_ (*e*; purple trace), and AFB_1_ + Gö6983 (*e*; green trace). Normalized CBF was 0.99 ± 0.01, 1.0 ± 0.01, and 1.0 ± 0.01 after 5 min application of 0.001%, 0.01%, and 0.1% DMSO, respectively. After 5 min application of AFB_2_, CBF decreased to 0.94 ± 0.01 (0.1 μM AFB_2_; *n.s*. compared with vehicle), 0.90 ± 0.02 (1 μM AFB_2_; *P* < 0.05 compared with vehicle) and 0.87 ± 0.02 (10 μM AFB_2_; *P* < 0.01 vs vehicle). In the presence of Gö6983, CBF with AFB_2_ was 0.99 ± 0.01 (0.1 μM AFB_2_), 0.96 ± 0.02 (1 μM AFB_2_), and 0.96 ± 0.03 (10 μM AFB_2_; *P* < 0.05 vs 10 μM AFB_2_ alone; *n.s*. vs vehicle) after 5 minutes. With AFB_1_, CBF decreased to 0.96 ± 0.01 (0.1 μM AFB_1_; *n.s*. compared with vehicle), 0.92 ± 0.01 (1 μM AFB_1_; *P* < 0.05 vs. vehicle), and 0.89 ± 0.01 (10 μM AFB_1_; *P* < 0.05 vs. vehicle). With AFB_1_ in the presence of Gö6983, CBF was 0.99 ± 0.01 (0.1 μM AFB_1_; *n.s*. compared with vehicle), 0.98 ± 0.01 (1 μM AFB_1_; *n.s*. compared with vehicle) and 0.98 ± 0.02 (10 μM AFB_1_; *n.s*. compared with vehicle). (**f**,**g**) Plot of Normalized CBF after 5 min vs log AFB_2_ (*f*) and AFB_1_ (*g*) Data points for 0.01 μM AFB_1_ and AFB_2_ were from separate experiments (not shown). Asterisks denote significance vs. DMSO alone (vehicle control). All significances determined by 1-way ANOVA with Bonferroni post-test; *P < 0.05, **P < 0.01. (**h**) Additive effects on CBF were not observed between the PKC-activator PMA (1 μM) and AFB_2_.

We examined epithelial responses to a common aflatoxin, aflatoxin B_2_ (AFB_2_). AFB_2_ (Fig. 1a) was chosen as the model aflatoxin for testing in this study because it has less carcinogenicity than AFB_1_^34^,^35^ and thus should have less DNA-damaging nonspecific toxic effects. We utilized air-liquid interface cultures (ALIs) derived from human sinonasal and bronchial epithelial cells^36^. ALIs mimic the polarized respiratory epithelium with well differentiated ciliated and goblet cells^37^. High-speed imaging was used to track changes in CBF. Acute mucosal exposure (apical side only) of sinonasal ALIs to AFB_2_ (1 μM and 10 μM) significantly decreased basal CBF after only 5 minutes, while vehicle (DMSO) had no effect (Fig. 1b,c). The protein kinase C (PKC) inhibitor Gö6983^38^ (10 μM; 5 min apical pre-treatment before experiment) significantly blunted the AFB_2_-mediated inhibition (Fig. 1d). AFB_1_ had nearly identical effects (Fig. 1e). Results are summarized in Fig. 1f,g. No additive effects of AFB_2_ were observed when sinonasal ALIs were pre-treated with the phorbol ester phorbol-12-myristate-13-acetate (PMA), nor was CBF further reduced when PMA was added to ALIs pre-treated with AFB_2_ (Fig. 1h), supporting the hypothesis that AFB_2_ reduces CBF through a PKC-dependent pathway.

**Figure 2:**
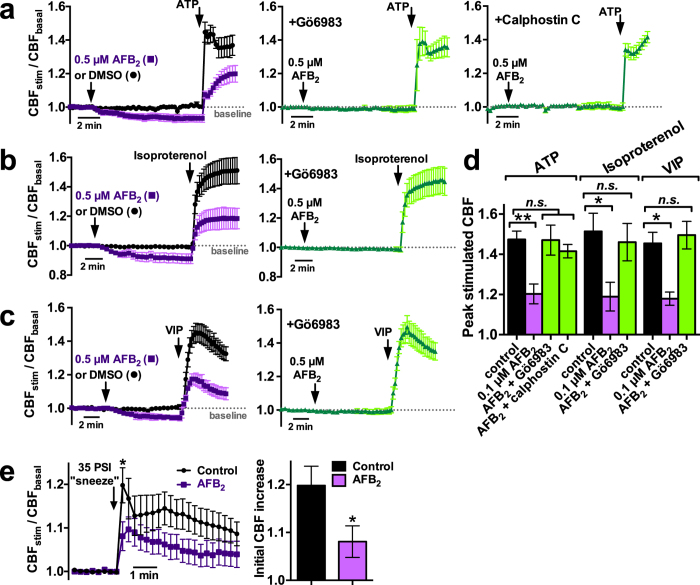
AFB_2_ decreased stimulated sinonasal CBF in a PKC-dependent manner. (**a–c**) Mean traces of CBF normalized to baseline (n = 3–6 cultures from separate patients each) during 10 min exposure to vehicle (DMSO), AFB_2_, or AFB_2_ + Go6983, or AFB_2_ + calphostin C, followed by subsequent stimulation with 1 μM ATP (*a*), 10 μM isoproterenol (*b*), or 10 μM VIP (*c*). Baseline CBF was only slightly reduced by AFB_2_ (0.93 ± 0.02; *P* < 0.05 vs. 1.00 ± 0.01 with DMSO; *P* < 0.05 vs. 0.99 ± 0.02 with AFB_2_ + Gö6983; P < 0.05 vs. 1.0 ± 0.02 with AFB_2_ + calphostin C). After subsequent stimulation with ATP (*a*), CBF increased to 1.48 ± 0.04 (DMSO), 1.20 ± 0.05 (AFB_2_; *P* < 0.01 vs DMSO), 1.47 ± 0.07 (AFB_2_ + Gö6983; *n.s*. vs DMSO), and 1.42 ± 0.03 (AFB_2_ + calphostin C; *n.s*. vs DMSO). After stimulation with isoproterenol (*b*), CBF increased to 1.51 ± 0.09 (DMSO), 1.19 ± 0.07 (AFB_2_; *P* < 0.05 vs DMSO), and 1.46 ± 0.09 (AFB_2_ + Gö6983; *n.s*. vs DMSO). After stimulation with VIP (*c*); CBF increased to 1.45 ± 0.05 (DMSO), 1.18 ± 0.03 (AFB_2_; *P* < 0.05 vs DMSO), and 1.50 ± 0.07 (AFB_2_ + Gö6983; *n.s*. vs DMSO). (**d**) Bar graph summarizing stimulated CBF data from *A-C*. Significances determined by 1-way ANOVA with Bonferroni post-test; *P < 0.05, **P < 0.01. (**e**) Trace of normalized CBF during a sneeze puff stimulus (left) and bar graph of CBF increase (right; 1.08 ± 0.03 with AFB_2_ vs 1.20 ± 0.04 with DMSO; *P* < 0.05 by Student’s *t* test).

We noted that short-term exposures to AFB_2_ and AFB_1_ also impaired activation of CBF in response to the purinergic agonist ATP (Fig. 1b,d), an important signaling molecule in the airway^33^. We thus carried out a detailed examination of the effects of AFB_2_ on stimulated CBF using several physiologically important agonists after 10 min exposure to 0.5 μM AFB_2_. AFB_2_ inhibited CBF during stimulation with 1 μM ATP (added apically; Fig. 2a), 10 μM isoproterenol (added apically; Fig. 2b), and 10 μM VIP (added basolaterally; Fig. 2c). CBF reductions (summarized in Fig. 2d) were blocked by the PKC inhibitors Gö6983 and calphostin C^38^. AFB_2_ exposure also reduced CBF increases in response to a mechanically-simulated “sneeze” (Fig. 2e), which stimulates CBF through apical ATP release and downstream calcium signaling^36^.

**Figure 3:**
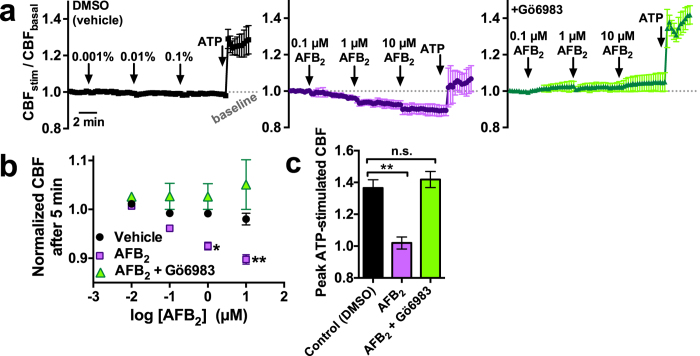
AFB_2_ also decreased basal and stimulated bronchial CBF. (**a**) Traces of normalized CBF during exposure to DMSO (vehicle control; left), AFB_2_ (middle), or AFB_2_ in the presence of Gö6983 (right) as well as during subsequent stimulation with 1 μM ATP. (**b**) Plot of baseline CBF vs log AFB_2_ concentration. Baseline CBF after 5 min with DMSO was 1.0 ± 0.01 (0.0001%; raw trace not shown), 0.99 ± 0.01 (0.001%), 0.99 ± 0.01 (0.01%), and 0.98 ± 0.01 (0.1%). Baseline CBF after 5 min with AFB_2_ was 1.0 ± 0.01 (0.01 μM; raw trace not shown; *n.s*. vs DMSO), 0.96 ± 0.004 (0.1 μM; n.s. vs DMSO), 0.93 ± 0.01 (1 μM; *P* < 0.05 vs DMSO), and 0.90 ± 0.01 (10 μM; *P* < 0.01 vs DMSO). Baseline CBF after 5 min with AFB_2_ in the presence of Gö6983 was 1.03 ± 0.003 (0.01 μM; raw trace not shown), 1.03 ± 0.03 (0.1 μM), 1.03 ± 0.03 (1 μM), and 1.05 ± 0.05 (10 μM; all values n.s. vs. DMSO). (**c**) Bar graph of peak ATP-stimulated CBF in the presence of DMSO (1.37 ± 0.05), AFB_2_ (1.02 ± 0.04; *P* < 0.01 vs DMSO), and AFB_2_ + Gö6983 (1.42 ± 0.05; n.s. vs DMSO). All significances determined by 1-way ANOVA with Bonferroni post-test; *P < 0.05, **P < 0.01.

When we examined ALIs grown from human bronchial epithelial (HBE) cells, we found that AFB_2_ similarly reduced both basal and ATP-stimulated CBF via a PKC-dependent mechanism (Fig. 3). Interestingly, when we examined ALI cultures derived from mouse nasal septum, we found that AFB_2_ inhibited basal CBF but not ATP-stimulated CBF (Supplementary Fig. S1), reflecting a species-specific difference.

### AFB_2_ Acts Independently of Calcium

Calcium is a master regulator in airway cells, controlling both ion transport^39^ as well as CBF^32^. Data above show that AFB_2_ reduces CBF in human ALIs in response to both ATP and the sneeze puff, which both require intracellular calcium, as well as VIP, which acts independently of calcium through cyclic AMP (cAMP) in these cells^40^. Thus, we hypothesized that AFB_2_ likely has direct effects on cilia function, possibly through PKC phosphorylation of cilia proteins, as previously described^33^, rather than by indirectly affecting calcium levels. However, because many isoforms of PKC are regulated by calcium^33^, we examined if AFB_2_ affects baseline or stimulated calcium signaling. We examined changes in intracellular calcium concentration in sinonasal ALIs loaded with the calcium-sensitive indicator fluo-4 during exposure to 10 μM AFB_2_. AFB_2_ had no detectible effect on intracellular calcium, nor did it affect the magnitude or kinetics of ATP-induced calcium signaling (Supplementary Fig. S2), supporting the hypothesis that AFB_2_ activates PKC independently of calcium.

### AFB_2_ Acts Independently of the Y_2_ Neuropeptide Y Receptor

**Figure 4:**
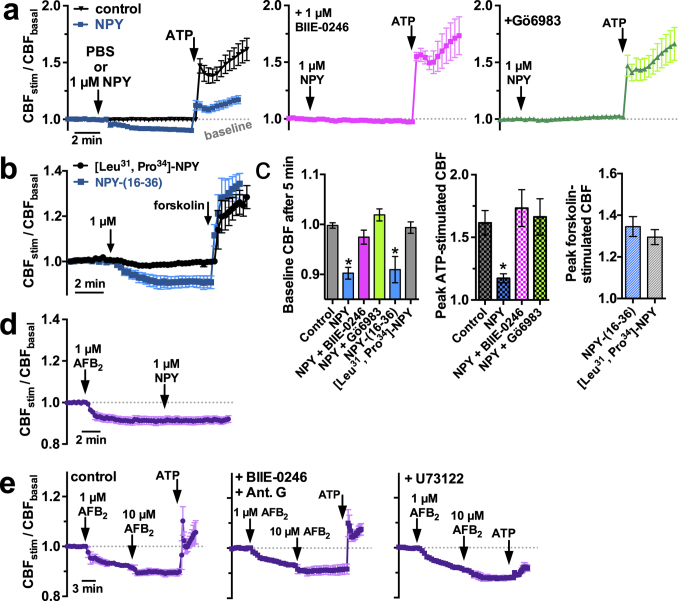
AFB_2_ acts independently of the NPY Y_2_ receptor. (**a**) Average traces (4–6 cultures from at least 3 patients for each condition) of CBF in sinonasal ALIs exposed to PBS (vehicle; left; black trace), 1 μM NPY (left; blue trace), or NPY in the presence of BIIE-0246 (5 μM; middle magenta trace) or Gö6983 (right green trace), followed by subsequent 10 μM ATP stimulation. (**b**) Average CBF trances (4–6 cultures from at least 3 patients for each condition) of sinonasal ALIs stimulated with [Leu^31^,Pro^34^]-NPY (black) and NPY-(16–36) (blue). (**c**) Left, bar graph showing baseline CBF after 5 min under control (PBS) conditions (1.0 ± 0.01), with NPY (0.90 ± 0.01; P < 0.05 vs control), NPY + BIIE-0246 (0.97 ± 0.01; n.s. vs. control), NPY + Gö6983 (1.02 ± 0.01; n.s. vs. control), NPY-(16–36) (0.91 ± 0.03; P < 0.05 vs control), and [Leu^31^,Pro^34^]-NPY (0.99 ± 0.01; n.s. vs. control). Middle, bar graph showing peak ATP stimulated CBF with vehicle (1.61 ± 0.1), NPY (1.17 ± 0.04; P < 0.05 vs control), NPY + BIIE-0246 (1.73 ± 0.15; n.s. vs. control) and NPY + Gö6983 (1.67 ± 0.14; n.s. vs. control). Right, bar graph showing peak CBF during forskolin stimulation with NPY-(16–36) (1.35 ± 0.05) and [Leu^31^,Pro^34^]-NPY and (1.20 ± 0.04; n.s.). (**d**) Average CBF trace showing sequential addition of AFB_2_ followed by NPY. (**e**) Average CBF traces showing CBF changes in response to 1 and 10 μM AFB_2_ followed by 1 μM ATP under control conditions (left) and in the presence of 10 μM BIIE-0246 and 10 μM antagonist G (middle) or 100 μM U73122 (right). Significances determined by 1-way ANOVA with Dunnett’s post test; *P < 0.05 vs control.

Neuropeptide Y (NPY) is one of the few neurotransmitters known to reduce CBF through Y_2_ receptor activation of PKC in primary human tracheal and bronchial ciliated cells^41^. In sinonasal ALIs, NPY decreased basal CBF by ~10% through a mechanism blocked by both the Y_2_ antagonist BIIE-0246 and Gö6983 (Fig. 4a,c). CBF was also reduced by the Y_2_ agonist NPY-(16–36) but not the Y_1_ agonist [Leu^31^,Pro^34^]-NPY (Fig. 4b,c). No additive effects were observed when NPY was added after AFB_2_ (Fig. 4d), suggesting they partially share the same pathway. However AFB_2_ reduction of CBF was not blocked by BIIE-0246, the broad spectrum neuropeptide receptor inhibitor antagonist G^42^, or the phospholipase C inhibitor U73122 (Fig. 4e). These data demonstrate that AFB_2_ functions independently of the Y_2_ receptor and likely other neurotransmitter receptors.

### Exposure to *A. flavus* conditioned media (CM) reduces CBF via a PKC- and aflatoxin-dependent pathway

**Figure 5:**
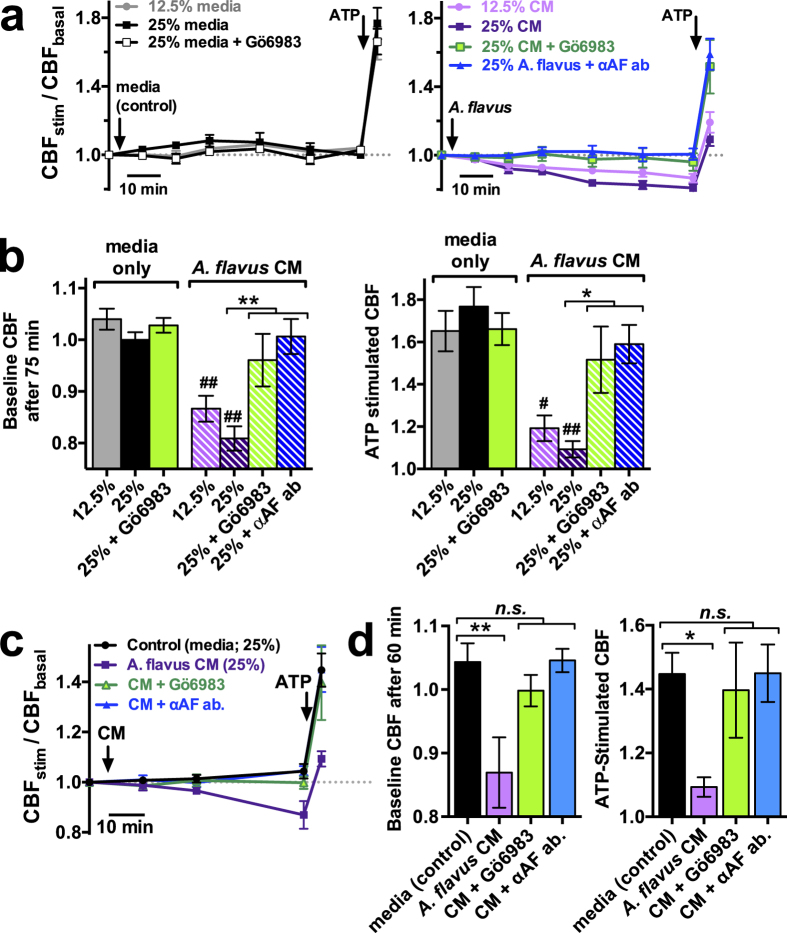
*A. flavus* conditioned medium (CM) reduces basal and stimulated CBF in a PKC-dependent and aflatoxin-dependent manner. (**a**) Average measurements of basal and ATP-stimulated sinonasal CBF (10 random fields from 4–5 cultures each per timepoint) in the presence of media only (left) or *A. flavus* CM (right). (**b**) Bar graph of baseline CBF after 75 min (left) and CBF immediately after ATP stimulation (right). Baseline CBFs were 1.04 ± 0.02 (12.5% media), 1.00 ± 0.01 (25% media), 1.03 ± 0.01 (25% media + Gö6983), 0.87 ± 0.03 (12.5% CM), 0.81 ± 0.02 (25% CM), 0.96 ± 0.05 (25% CM + Gö6983), and 1.01 ± 0.03 (25% CM + anti-aflatoxin [αAF] antibody). Statistical significance indicated in the figure determined by 1-way ANOVA with Bonferroni post-test; ^#^P < 0.05 and ^##^P < 0.01 vs. control (media only), *P < 0.05 and **P < 0.01 vs. bracketed set. (**c**) Average measurements of basal and ATP-stimulated CBF as in *A* but with bronchial ALIs. (**d**) Bar graph showing baseline (left) and ATP-stimulated (right) CBF. Baseline CBFs were 1.04 ± 0.03 (25% media), 0.87 ± 0.06 (25% CM), 0.99, ± 0.02 (25% CM + Gö6983), and 1.05 ± 0.02 (25% CM + αAF antibody). ATP stimulated CBFs were 1.45 ± 0.07 (25% media), 1.09 ± 0.03 (25% CM), 1.40, ± 0.15 (25% CM + Gö6983), and 1.45 ± 0.09 (25% CM + αAF antibody). Significance determined by 1-way ANOVA with Bonferroni post test; *P < 0.05 and **P < 0.01 vs. bracketed set.

We tested whether conditioned medium (CM) from a known aflatoxin-producing strain of *A. flavus* could similarly reduce respiratory CBF. Experiments were carried out in sinonasal ALIs at 12.5% and 25% (Fig. 5a,b) and confirmed in bronchial ALIs at 25% (Fig. 5c,d). *A. flavus* CM significantly reduced baseline CBF after 60–75 min and significantly blunted ATP-induced CBF increase (Fig. 5b,d). While the kinetics of the CM-induced reduction in CBF was slower than observed with purified aflatoxin, these effects were nonetheless blocked by Gö6983 as well as when the *A. flavus* CM was pre-treated with anti-aflatoxin antibodies (recognizing both B and G group aflatoxins). These data strongly suggest that cultured *A. flavus* secretes aflatoxins at low concentrations that are nonetheless high enough to reduce airway CBF. We observed that CM from *A. fumigatus* and *A. niger*, which cannot secrete aflatoxins, was still observed to reduce CBF (Supplementary Fig. S3). The effects of *A. fumigatus* and *A. niger* CM were blocked by Gö6983 but not by anti-aflatoxin antibodies (Supplementary Fig. S3), suggesting that these species secrete other mycotoxins that can target PKC, perhaps including gliotoxin, fumagillin, and/or helvoilic acid. The identities and mechanisms of action of *A. fumigatus* and *A. niger* ciliotoxins remain to be determined in future studies.

### AFB_2_ impairs sinonasal epithelial nitric oxide (NO) innate immune responses

**Figure 6:**
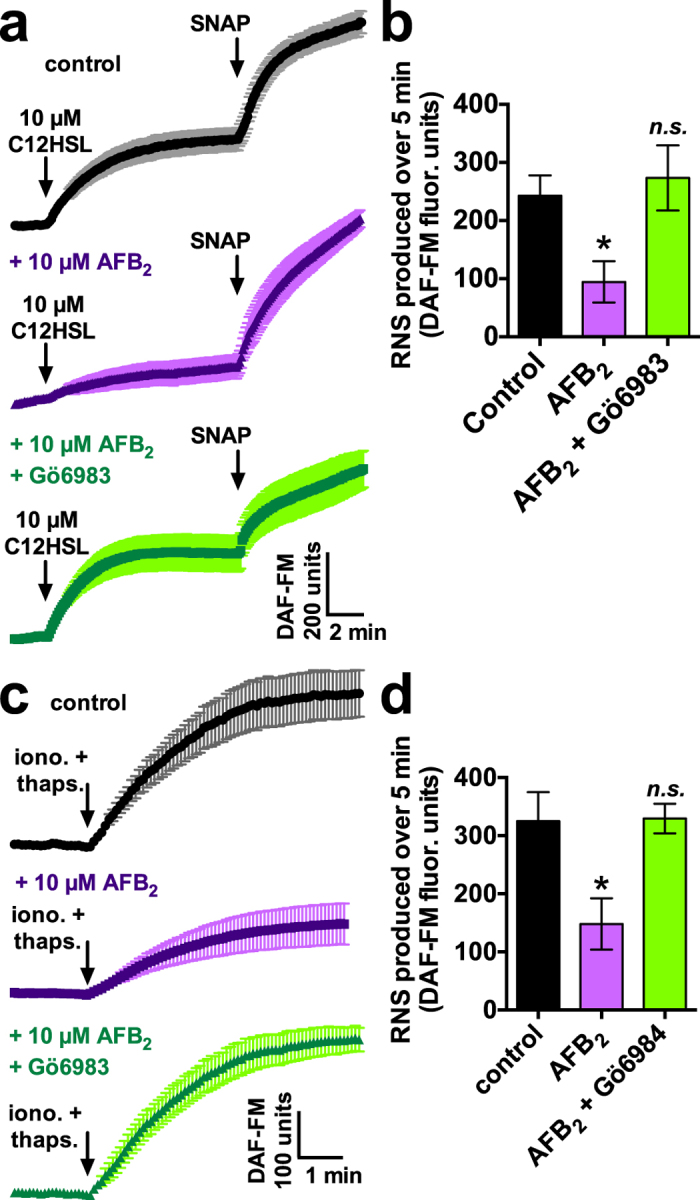
AFB_2_ reduces sinonasal epithelial NO production in response to the *P. aeruginosa* quorum-sensing molecule and T2R38 receptor agonist C12HSL. (**a**) Average traces of RNS production (DAF-FM fluorescence) during stimulation of sinonasal epithelial cells (n = 3–4 cultures from 2 genotyped PAV/PAV [functional T2R38^43^] patients each) with 10 μM C12HSL under control (vehicle) conditions as well as after exposure to 10 μM AFB_2_ ± Gö6983 followed by exposure to S-Nitroso-N-Acetyl-D,L-Penicillamine (SNAP), a non-specific NO donor. (**b**) Bar graph of NO production in response to C12HSL in the presence of vehicle (243 ± 35 DAF-FM fluorescence units), AFB_2_ (95 ± 36 units; *P* < 0.05 vs vehicle) or AFB_2_ + Gö6983 (274 ± 56 units; n.s. vs vehicle). (**c**) Average traces of RNS production (DAF-FM fluorescence) during exposure of sinonasal epithelial cells (n = 4–6 cultures per condition) exposed to 10 μM ionomycin and 10 μM thapsigargin under control conditions as well as after exposure to 10 μM AFB_2_ ± Gö6983. (**d**) Bar graph of NO production from *C* in the presence of vehicle (325 ± 50), AFB_2_ (148 ± 44; *P* < 0.05 vs vehicle) or AFB_2_ + Gö6983 (330 ± 25 units; n.s. vs vehicle). Significances determined by 1-way ANOVA with Dunnett’s post test; *P < 0.05 vs control.

Nitric oxide (NO) is an important mediator of host airway defense because it directly kills pathogens as well as increases CBF^43^,^44^. We recently showed that a bitter taste receptor, T2R38, is expressed in sinonasal epithelial cilia and drives NO production in response to bacterial acyl-homoserine lactone (AHL) quorum sensing molecules^43^,^44^,^45^. Because PKC can phosphorylate nitric oxide synthase (NOS) and prevent its activation^46^,^47^, we tested the effects of AFB_2_ on sinonasal NO production in response to the T2R38 agonist and *Pseudomonas* quorum sensing molecule N-3-oxo-dodecanoyl-L-homoserine lactone (C12HSL)^43^. Reactive nitrogen species (RNS) production was measured using the fluorescent indicator DAF-FM. RNS production was reduced by approximately one half in the presence of AFB_2_, and this effect was blocked by Gö6983 (Fig. 6a,b). To test if AB_2_-induced PKC activity had a general effect on NOS function or a specific effect on T2R38 function, we measured RNS production during global calcium elevation in cells exposed to the calcium ionophore ionomycin (10 μM) and the sarco/endoplasmic reticulum calcium ATPase (SERCA) inhibitor thapsigargin (10 μM). AFB_2_ also significantly reduced NO production under these conditions through a Gö6983-sensitive pathway (Fig. 6c,d), suggesting AFB_2_ has a direct effect on NOS activation rather than on T2R38 function.

### AFB_2_ activates PKC in A549 cells *in vitro*

To further test the hypothesis that aflatoxins can activate PKC activity, we utilized a Förster resonance energy transfer (FRET)-based PKC construct, CKAR^48^,^49^. Because AFs activate PKC in a variety of cell types^29^,^30^,^31^,^50^, we hypothesized that the mechanism of activation was not cell-type dependent. As primary sinonasal ALIs are very difficult to transfect, even with viral systems, CKAR was transfected into A549 cells, a commonly used lung epithelial cell line. CKAR contains the FHA2 domain of RAD53p as well as a PKC phosphorylation sequence designed to be phosphorylated by all PKC isoforms. These sequences are flanked by an eCFP the N-terminus and a citrine YFP variant on the C-terminus. When phosphorylated, the substrate sequence binds the FHA2 phospho-peptide-binding domain, resulting in a conformational change that keeps the CFP and YFP further apart, reducing FRET emission. Thus, a decrease in FRET emission correlates with an increase in PKC activity, and vice verse. This conformational change is reversible by phosphatases. Single transfected cells were imaged by conventional wide-field low-light-level microscopy, collecting light at three wavelengths: 1) CFP excitation, CFP emission, 2) CFP excitation, YFP emission, and 3) YFP excitation, YFP emission. Data are reported as the signal of wavelength 2 divided by wavelength 1 (i.e. the yellow/cyan emission ratio at cyan excitation, or FRET/CFP ratio) as previously described^48^.

**Figure 7:**
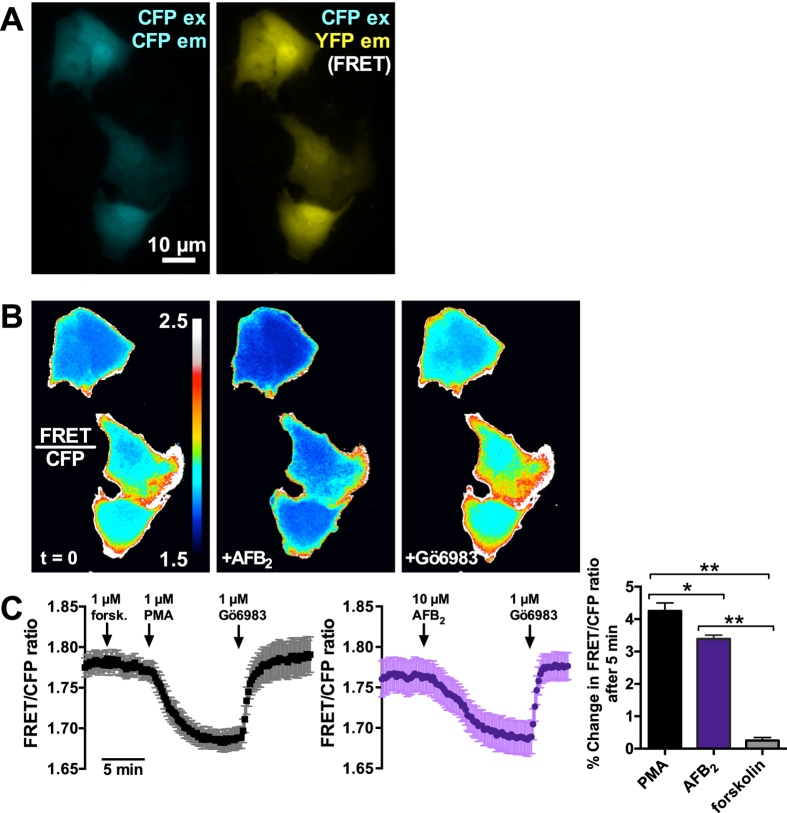
AFB_2_ activates PKC in A549 cells *in vitro*. (**a**) Representative image of A549 cells transfected with CKAR, showing CFP (left) and FRET (right) signals (**b**) Ratiometric images showing CFP/FRET ratio at baseline (left panel), after stimulation with 10 μM AFB_2_ (middle panel), and after subsequent 1 μM Gö6983. (**c**) Average traces (mean ± SEM) of CKAR FRET/CFP ratio during stimulation with forskolin and PMA (left) and AFB_2_. Traces are the average of 4 (forskolin/PMA) and 9 (AFB_2_) experiments. Bar graph to the right shows % change in CKAR FRET/CFP ratio, which was 4.3 ± 0.2% with PMA, 3.4 ± 0.1% with AFB2, and 0.2 ± 0.09% with forskolin. Significances determined by 1-way ANOVA with Bonferroni post test; *P < 0.05 vs control.

Application of 10 μM AFB_2_ caused a decrease in CKAR FRET/CFP ratio that was reversible by addition of Gö6983 in the continued presence of AFB_2_ (Fig. 7a–c). As a control, A549 CKAR-transfected cells treated with PMA exhibited a fast decrease in CKR FRET that was reversed with application of Gö6983 (1 μM) in the continued presence of PMA (Fig. 7c). Application of forskolin, an activator of adenylate cyclase, had no effect on CKAR fluorescence, as previously described^48^ (Fig. 7c). These results strongly support the hypothesis that AFB_2_ exposure increases PKC activity.

## Discussion

The average person inhales hundreds to thousands of airborne *Aspergillus* spores daily^10^. In immune-competent individuals, these fungi are typically cleared without consequence. However, in individuals with impaired respiratory defenses (e.g., patients with CRS, diabetes, CF or otherwise immunocompromised), fungal infection can be a significant or even a fatal complication^9^. Understanding the effects of mycotoxins on the respiratory epithelium is important for understanding the pathogenesis of respiratory (upper and lower) aspergillosis. Here, we show that a class of *Aspergillus* mycotoxins, aflatoxins, can slow basal and stimulated respiratory CBF, potentially enhancing *A. flavus* pathogenesis by impairing MCC. Aflatoxins target PKC, previously shown in other studies to slow CBF^33^. This occurs without alteration of calcium or baseline NO signaling.

The affects observed here are in response to acute aflatoxin exposure. Longer-term studies of the effects of aflatoxins on mucociliary transport, potentially in an animal model of aflatoxin-exposure, will help to shed light on situations of chronic exposure and effects on airways. To our knowledge, studies of longer term exposures have only been previously done in airway cells^34^,^51^,^52^,^53^,^54^,^55^,^56^,^57^ and animals^28^,^58^,^59^,^60^,^61^,^62^ with a focus on carcinogenesis. The concentrations of aflatoxins used here (0.1–10 μM) are in the same range used in previous *in vitro* and *ex vivo* studies by other groups^4^,^52^,^53^,^56^,^57^. However, it must also be determined how environmental aflatoxin exposure, often measured in ppm of aflatoxin-contaminated dust, actually translates to concentrations seen by the airway epithelial cells. As exposure often occurs through contaminated dust, airway deposition will be affected by particle size and sinonasal airflow patterns^63^. This would be further confounded by the fact that the most commonly used animal models, such as mice, have significantly different paranasal sinus anatomy than humans^64^,^65^. Sampling of airway surface liquid and mucus from patients with respiratory *A. flavus* infections may shed light on concentrations of aflatoxins generated during active A. flavus infection. Moreover, since a significant amount of aflatoxin contamination occurs in grain-based livestock and pet foods^66^,^67^,^68^, inhalation of aflatoxin-contaminated dust may also be a contributor to respiratory infection in non-human animals as well. Antibiotic use in animals is a major driver for the emergence of resistant pathogenic microorganisms^69^. Further studies of aflatoxin exposure levels in at-risk humans and animal models are critically needed to help complete our understanding of the consequences of both acute and chronic aflatoxin respiratory exposure in humans an animals.

Coupled with previous data that *Aspergillus* gliotoxin, fumagillin, and helvoilic acid^17^,^18^ slow CBF, the current data emphasize that *Aspergillus* have evolved an armament of mycotoxins to impair MCC and reduce host innate defense. Our data also show that *A. niger* and *A. fumigatus*, which cannot secrete aflatoxins, nevertheless secrete mycotoxins that also activate PKC. Certain PKC isoforms have also been linked to inflammation^70^ and apoptosis^71^, and thus chronic exposure to aflatoxins and other *Aspergillus* mycoctoxins may stimulate these processes, exacerbating epithelial damage. PKC inhibitors have been proposed as therapeutics for inflammatory diseases^70^. Our study suggests that PKC inhibitors may also have potential for fungal airway diseases by relieving mycotoxin-induced repression of ciliary beating. Moreover, the ability of AFB_2_ to impair sinonasal NO production in response to bacterial AHL-stimulation of T2R receptors suggests that aflatoxins may play an important role in the generation of mixed fungal and bacterial biofilms sometimes observed in airway diseases^72^. Because we have shown that reduced T2R38 function correlates with gram-negative bacterial infection^43^, risk of chronic rhinosinusitis^73^,^74^, and surgical outcomes in non-polypoid chronic rhinosinusitis^75^, exposure to aflatoxins and resulting reduction in downstream components of the T2R38 pathway may have important implications for all of these clinical parameters. Moreover, the ability of aflatoxins to impair ciliary activity may have likewise profound clinical consequences during pulmonary aspergillosis caused by *A. flavus*^76^.

In conclusion, exposure of ciliated respiratory epithelial cells to AFB_2_ resulted in a decrease in both baseline and stimulated CBF through calcium-independent activation of PKC. AFB_2_ also impaired sinonasal epithelial cell bitter taste receptor-driven NO innate immune responses to gram-negative bacterial quorum sensing molecules. These results suggest that aflatoxins may impair MCC and other innate defense pathways, enhancing the pathogenicity of *A. flavus* and possibly other co-infecting pathogens as well. In addition to their anti-inflammatory effects, PKC inhibitors may be potential therapeutics for fungal respiratory diseases due to their ability to counteract mycotoxin-induced decreases in ciliary beating and MCC.

## Materials and Methods

All experimental protocols were reviewed and approved by the Research and Development Committee at the Philadelphia Veterans Affairs Medical Center and were carried out in accordance with both The University of Pennsylvania and The Philadelphia VA Medical Center guidelines regarding use of residual clinical material in research.

### Reagents and solutions

Unless indicated, all reagents and solutions were as previously described^40^,^43^,^77^,^78^. Fluo-4 and DAF-FM were from Invitrogen (Grand Island, NY). Aflatoxins B_1_ (AFB_1_) and B_2_ (AFB_2_) were from Cayman (Ann Arbor, MI). Gö6983, BIIE-0246, [Leu^31^,Pro^34^]-NPY, NPY-(16–36), antagonist G, and calphostin C were from Tocris (Minneapolis, MN USA). All other reagents were from Sigma-Aldrich (St. Louis, MO USA). Stock solutions of aflatoxin were 10 mM in DMSO. Working solutions (10, 1, and 0.1 μM) contained 0.1%, 0.01%, and 0.001% DMSO, respectively, and were made up immediately before use; activities of aqueous solutions were observed to be markedly reduced after ~1–2 hr at room temp. Anti-aflatoxin antibodies (recognizing AFB_1_, AFB_2_, and AFG) were from Thermo Scientific (MA-7386) and Sigma Aldrich (A9555).

### Generation of sinonasal ALI cultures

Patients undergoing sinonasal surgery were recruited from the Department of Otorhinolaryngology at the University of Pennsylvania and the Philadelphia Veterans Affairs Medical Center with full approval of both Institutional Review Boards (Penn#800614, PVAMC#00781) and written informed consent was obtained for all participating patients in accordance with the U.S. Department of Health and Human Services code of federal regulation Title 45 CFR 46.116. Exclusion criteria included a history of systemic diseases (e.g., Wegner’s, Sarcoid, CF), immunodeficiences, or use of antibiotics, oral corticosteroids, or anti-biologics (e.g. Xolair) within one month of surgery. Human sinonasal epithelial cells were enzymatically dissociated grown to confluence in proliferation medium (DMEM/Ham’s F-12 plus BEBM; Clonetics, Cambrex, East, NJ, USA) for 7 days as previously described^37^,^43^. Confluent cells were dissociated and seeded on porous polyester membranes coated with BSA, type I bovine collagen, and fibronectin in cell culture inserts in LHC basal medium (Invitrogen). Culture medium was removed from the upper compartment and basolateral media was changed to differentiation medium (1:1 DMEM:BEBM) containing hEGF (0.5 ng/ ml), epinephrine (5 g/ml), BPE (0.13 mg/ml), hydrocortisone (0.5 g/ml), insulin (5 g/ml), triiodothyronine (6.5 g/ml), and transferrin (0.5 g/ml), supplemented with 100 U/ml penicillin, 100 g/ml streptomycin, 0.1 nM retinoic acid, and NuSerum (BD Biosciences, San Jose, CA) as previously described^37^,^43^.

### Measurement of ciliary beat frequency (CBF)

Whole-field CBF was measured using the Sisson-Ammons Video Analysis system^79^ as previously described^36^,^40^,^43^ at ~28–30 °C. Cultures were imaged using at 100 frames/second using a Leica Microscope (20x/0.8NA objective) with Hoffman modulation contrast. Experiments utilized Dulbecco’s PBS (1.8 mM calcium) on the apical side and HEPES-buffered Hank’s Balanced Salt Solution supplemented with 1× MEM vitamins and amino acids on the basolateral side.

### Calcium and nitric oxide (NO) imaging

Calcium and NO were imaged using the Fluo-4 and DAF-FM, respectively, as previously described^36^,^43^,^77^,^78^. Cultures were loaded with Fluo-4 AM (10 μM applied apically) for 2 hrs followed by washing and 20 min incubation in the dark. Cultures were similarly loaded with 10 μM DAF-FM diacetate for 90 min in the presence of 5 μM carboxy-PTIO, followed by washing to remove unloaded DAF-FM and cPTIO and incubation for 15 minutes prior to imaging. Imaging was performed using an Olympus Fluoview confocal system with IX-81 microscope and 10x (0.3 NA UPlanFLN) objective. Images were analyzed using Fluoview software as previously described^36^,^43^,^77^,^78^. Fluo-4 fluorescence was normalized after subtraction of background, estimated by unloaded ALIs at identical settings. Baseline fluorescence (F_o_) was determined from the first 10 frames of each experiment. DAF-FM measurements utilized raw fluorescence values to compare experiments performed under identical conditions and settings.

### Fungal culture

Cultures of *A. niger* (strain WB326 [ATCC16888] and a clinical isolate from the Philadelphia VA Medical Center), *A. fumigatus* (NIH5233 [ATCC 13073] and NRRL163 [ATCC1022]), and *A. flavus* (NRRL3357 [ATCC200026]) were grown in 40 ml BACTEC Myco/F Lytic Culture Vials (BD, Sparks, MD) with assistance from the Philadelphia VA clinical microbiology lab. Inoculated cultures were grown at 30 °C for ten days. Conditioned medium (CM) was extracted and filtered sequentially through 0.45 μm and 0.2 μm filters.

### A549 cell culture, transfection, and CKAR FRET imaging

A549 cells were obtained from American Type Culture Collection (ATCC, Manassas, VA) and cultured in Kaighn’s modification of Ham’s F12 media (F12K) with 10% fetal bovine serum and 1x penicillin-streptomycin mix (Gibco/Thermo Fisher Scientific, Waltham, MA). Cells were used at passage 15–20. Cells were transfected with CKAR^48^,^49^ (Alexandra Newton, University of California San Diego, Addgene, Cambridge, MA, plasmid#14860) by standard calcium phosphate transfection in a 150 mm dish at ~75% confluency. The day after transfection, cells were trypsinized and re-plated into chambered coverglass wells (CellVis, Mountain View, CA) at 50% confluency. Cells were used at 48 hrs after transfection, and cells media was replaced with 10 mM HEPES-buffered Hank’s balanced salt solution (HBSS) and imaging was performed at room temperature on the stage of an Olympus IX-83 inverted microscope (60x PlanApo 1.4 NA oil-immersion objective; Olympus Life Sciences, Tokyo, Japan) equipped with excitation and emission filter wheels (Sutter Instruments, Novato, CA) and a CFP-YFP FRET filter set (89003-ET, Chroma Technologies, Rockingham, VT). Images were acquired (12 sec intervals) and analyzed using MetaFluor (Molecular Devices, Sunnyvale, CA) and ratio images were constructed using ImageJ (W. Rasband, National Institutes of Mental Health, Research Services Branch, Bethesda, MD). Both background (estimated using an off-cell area) and baseline drift were subtracted as described^49^ before averaging of traces.

### Data analysis and statistics

One-way analysis of variance (ANOVA) was performed in GraphPad Prism with appropriate post-tests as indicated; *P* < 0.05 was considered statistically significant. All other data analysis was performed in Excel. For all figures, one (*) and two (**) asterisks indicate *P* < 0.05 and P < 0.01 respectively; “*n.s*.” indicates no statistical significance. All data are mean ± SEM.

## Additional Information

**How to cite this article**: Lee, R. J. *et al*. Fungal Aflatoxins Reduce Respiratory Mucosal Ciliary Function. *Sci. Rep*. **6**, 33221; doi: 10.1038/srep33221 (2016).

## Supplementary Material

Supplementary Information
