# Supplementary material for: Fungal Aflatoxins Reduce Respiratory Mucosal Ciliary Function
Source: Sci Rep. 2016 Sep 14;6:33221. doi: 10.1038/srep33221 (PMC5021939; doi:10.1038/srep33221)
Supplement: Supplementary Information [file srep33221-s1.doc]

**Supplementary Material:**

**Fungal Aflatoxins Reduce Respiratory Mucosal Ciliary Function**

Robert J. Lee^1,2^, Alan D. Workman^1^, Ryan M. Carey^1^, Bei Chen^1^, Phillip L. Rosen^1^, Laurel Doghramji^1^, Nithin D. Adappa^1^, James N. Palmer^1^, David W. Kennedy^1^, and Noam A. Cohen^1,3,4 *^

**Supplementary Figure S1. AFB_2_ reduced baseline but not stimulated CBF in mouse nasal septal ALI cultures.** **(a)** Normalized traces of CBF (4-5 ALIs for each condition) during exposure to DMSO (vehicle control), 10 µM AFB2, or AFB2 in the presence AFB_2_ + Gö6983, followed by subsequent stimulation with 1 µM ATP. **(b)** Bar graph of baseline CBFs after 5 minutes, which were 0.99 ± 0.02 (vehicle), 0.84 ± 0.06 (AFB_2_; *P* <0.05 vs. DMSO), 1.03 ± 0.02 (AFB_2_ + Gö6983, *n.s.* vs. DMSO). **(c)** Bar graph of peak ATP-stimulated CBFs, which were 1.84 ± 0.07 (vehicle), 2.14 ± 0.14 (AFB_2_, *n.s.* vs. DMSO), 1.89 ± 0.14 (AFB_2_, *n.s.* vs. DMSO). All significances determined by 1-way ANOVA with Dunnett’s post test; **P* <0.05 vs control.

**Supplementary Figure S2. AFB_2_ did not affect baseline calcium or stimulated calcium responses. (a)** Normalized traces of Fluo-4 showing intracellular calcium concentrations during stimulation with 10 µm AFB_2_ and 1 µM ATP.  **(b)**  Bar graphs showing magnitude and kinetics of the ATP-induced calcium responses after stimulation with vehicle and AFB_2_. Peak calcium responses with 1 µM ATP (reported as normalized Fluo-4 fluorescence) were 2.96 ± 0.22 (vehicle) and 2.86 ± 0.20 (AFB_2_). Times to peak calcium responses were 9 ± 1 sec (vehicle) and 8 ± 1 sec (AFB_2_). Times to 50% decay of the calcium peaks were 137 ± 15 sec (vehicle) and 117 ± 14 sec (AFB_2_). Significance determined by Student’s *t* test; *n.s.* = no statistical significance.

**Supplementary Figure S3. Aflatoxin-independent CBF reductions in sinonasal ALIs exposed to *A. fumigatus* and *A. niger* CM, but not *C. albicans* CM.** **(a-d)** Average measurements of basal and ATP-stimulated sinonasal CBF (10 random fields from 4-5 cultures each per timepoint) in the presence of media only (*a*) or CM from two strains of A. niger (*b*), two strains of A. fumagatus (*c*), and one strain C. albicans (*d*) at two concentrations and in the presence and absence of Gö6983 and an anti-aflatoxin antibody (α-AF ab). **(e)** Bar graph of basal CBF after exposure from experiments as in *a-d*. **(f)** Bar graph of ATP stimulated CBF from experiments as in *a-d*. CM from all A. fumagatus and A. niger has significant effects on baseline and ATP-stimulated CBF, which were blocked by Gö6983 but not aflatoxin antibody. CM from C. albicans had no detectible effects on CBF. All significances determined by 1-way ANOVA with Bonferroni post test; *P<0.05 and ***P* <001 vs, media at the same concentration.

**Supplementary Figure S4. Neither AFB_2_ nor *Aspergillus* conditioned media (CM) stimulated nitric oxide (NO) production in sinonasal epithelial cells. (a)** Average trace of reactive nitrogen species (RNS) production (DAF-FM fluorescence) during stimulation with AFB_2_ and subsequently during addition of the non-specific NO donor S-nitroso-N-acetyl-D,L-penicillamine (SNAP; n = 6 cultures from 3 different patients). **(b)** Bar graph showing RNS production in response to A. fumgatus, A. niger, A. flavus, and C. albicans CM or control media (CM) as indicated from experiments carried out as in *A*. CM had no significant effect on RNS production compared with media alone; ***P* <0.01 vs media control.
